# HDAC6 inhibition reverses long-term doxorubicin-induced cognitive dysfunction by restoring microglia homeostasis and synaptic integrity

**DOI:** 10.7150/thno.67410

**Published:** 2022-01-01

**Authors:** Blake R McAlpin, Rajasekaran Mahalingam, Anand K Singh, Shruti Dharmaraj, Taylor T Chrisikos, Nabila Boukelmoune, Annemieke Kavelaars, Cobi J Heijnen

**Affiliations:** 1 Laboratories of Neuroimmunology, Department of Symptom Research, Division of Internal Medicine, The University of Texas MD Anderson Cancer Center, Houston, Texas, USA.; 2 Department of Immunology, The University of Texas MD Anderson Cancer Center, Houston, Texas, USA.

**Keywords:** chemotherapy-induced cognitive dysfunction, HDAC6 inhibition, microglia, chemobrain, single-nucleus RNA sequencing

## Abstract

Breast cancer is the most common female malignancy in both the developed and developing world. Doxorubicin is one of the most commonly used chemotherapies for breast cancer. Unfortunately, up to 60% of survivors report long-term chemotherapy-induced cognitive dysfunction (CICD) characterized by deficits in working memory, processing speed and executive function. Currently, no therapeutic standard for treating CICD exists. Here, we hypothesized that treatment with a blood-brain barrier permeable histone deacetylase 6 (HDAC6) inhibitor can successfully reverse long-term doxorubicin-induced cognitive dysfunction.

**Methods**: The puzzle box test and novel object/place recognition test were used to assess cognitive function following a therapeutic doxorubicin dosing schedule in female mice. Mitochondrial function and morphology in neuronal synaptosomes were evaluated using the Seahorse XF24 extracellular flux analyzer and transmission electron microscopy, respectively. Hippocampal postsynaptic integrity was evaluated using immunofluorescence. Hippocampal microglia phenotype was determined using advanced imaging techniques and single-nucleus RNA sequencing.

**Results**: A 14-day treatment with a blood-brain barrier permeable HDAC6 inhibitor successfully reversed long-term CICD in the domains of executive function, working and spatial memory. No significant changes in mitochondrial function or morphology in neuronal synaptosomes were detected. Long-term CICD was associated with a decreased expression of postsynaptic PSD95 in the hippocampus. These changes were associated with decreased microglial ramification and alterations in the microglia transcriptome that suggest a stage 1 disease-associated microglia (DAM) phenotype. HDAC6 inhibition completely reversed these doxorubicin-induced alterations, indicating a restoration of microglial homeostasis.

**Conclusion**: Our results show that decreased postsynaptic integrity and a neurodegenerative microglia phenotype closely resembling stage 1 DAM microglia contribute to long-term CICD. Moreover, HDAC6 inhibition shows promise as an efficacious pharmaceutical intervention to alleviate CICD and improve quality of life of breast cancer survivors.

## Introduction

Breast cancer accounts for 12% of all new annual cancer cases in the developed and developing world, making it the most common female malignancy worldwide. Although the success of chemotherapy has increased the survival rate of breast cancer patients by 38% since 1989, up to 60% of survivors report persistent chemotherapy-induced neurotoxic side effects characterized by deficits in working memory, processing speed, executive function, and attention known collectively as chemotherapy-induced cognitive dysfunction (CICD) 1, 2. In long-term breast cancer survivors, CICD has been reported to persist up to 5 years after cessation of treatment 3. CICD is a debilitating impairment that causes emotional and financial burdens, significantly lowering the quality of life of cancer survivors. Currently, no intervention has been approved by the United States Food and Drug Administration to prevent or reverse CICD. We developed a therapeutic dosing schedule of doxorubicin to model CICD in mice and investigate the behavioral, transcriptional and cellular alterations that sustain it. Then, we evaluated the therapeutic effects of pharmacological HDAC6 inhibition in reversing doxorubicin-induced alterations and restoring cognitive function.

Doxorubicin is one of the most effective chemotherapy treatments for breast cancer patients. Doxorubicin's efficacy is associated with systemic inflammation and oxidative stress in the periphery, but its effects on the central nervous system (CNS) that initiate and sustain CICD are relatively unknown 4. Despite doxorubicin's poor penetration of the blood-brain barrier, recent pre-clinical research has associated neuroinflammation and doxorubicin-associated CICD. Immediately following dosing in rodents, doxorubicin treatment was shown to be correlated with an increase in pro-inflammatory cytokines TNF-α, IL-1β and IL-6 in the hippocampus and prefrontal cortex 5. Importantly, the release of pro-inflammatory cytokines in the hippocampus has been proposed to promote cognitive dysfunction 6. Pro-inflammatory cytokine release in the brain is mediated by the activation of glial cells, most notably astrocytes and microglia 7. Accordingly, microglia activation is associated with cognitive dysfunction in a variety of neurodegenerative disease models and during aging 8. However, the role of microglia in long-term CICD is unknown. Therefore, more evidence is needed to define microglia phenotype during doxorubicin-associated CICD long after cessation of treatment.

Changes in microglia phenotype are characterized by changes in gene expression and cellular morphology. Resting microglia have a ramified morphology, characterized by long, highly branched processes and, in situations of neuroinflammation, become de-ramified in a stepwise manner toward an activated state characterized by shortened, bushy processes 9. In addition to canonical microglia activation, alternative microglia phenotypes have recently been identified in aging and neurodegenerative disorders that are characterized by heightened immune reactivity, altered phagocytosis and persistent activation 10, 11. These microglia phenotypes have been called “primed”, “dystrophic” or “disease-associated microglia” (DAMs) depending on disease models 10, 12, 13. The role of these alternative microglia phenotypes in CICD is unknown. In general, these microglia phenotypes are characterized by a deramified morphology with shorter projections and less branching, comparable to the morphology of activated microglia 10. Due to similarities in morphology between canonically activated and alternative microglia phenotypes, additional genetic or molecular signatures are required to characterize and define the microglia phenotype. In the current study we characterized the microglia phenotype by analyzing microglial morphology and single-nucleus transcriptome in the hippocampus of doxorubicin-treated mice to investigate the mechanisms underlying long-term CICD.

Considering the close association between neuroinflammation and doxorubicin-associated CICD, pharmaceutical agents that target histone deacetylase 6 (HDAC6) may prove to be effective in alleviation of CICD. Inhibition of HDAC6, a class II histone deacetylase that resides in the cytoplasm, has recently been shown to attenuate microglia activation in mouse models of Parkinson's disease and suppress lipopolysaccharide (LPS)-induced neuroinflammation 14, 15. In neurodegenerative disease models of Alzheimer's and multiple sclerosis, HDAC6 inhibition improved cognitive dysfunction 16, 17. Importantly, we recently demonstrated that ACY-1083, a blood-brain barrier permeable HDAC6 inhibitor, successfully reversed cognitive dysfunction in a cisplatin-associated model of CICD by reversing deficits in synaptosomal mitochondrial bioenergetics 18.

As a cytoplasmic enzyme, HDAC6 does not interact directly with histones; its main substrates include heat shock protein 90 (HSP90) and α-tubulin, involved in protein folding and intracellular transport, respectively 19. Crucially, HDAC6 inhibitors have been shown to control breast cancer cell proliferation and migration both *in vitro* and *in vivo* 20-22. Ricolinostat (ACY-1215), an HDAC6 inhibitor, is currently being tested in multiple clinical trials to evaluate its enhanced tumor control in combination with chemotherapy (NCT01997840, NCT02787369, and NCT01583283). Due to the evidence that HDAC6 inhibition improves cognitive dysfunction in neurodegenerative models, attenuates neuroinflammation, and enhances tumor control, HDAC6 inhibitors may be an ideal candidate for pharmaceutical intervention of CICD.

Here, we tested the hypothesis that inhibition of HDAC6 with ACY-1083 reverses long-term CICD using a therapeutic dosing schedule of doxorubicin to model chemotherapy-induced cognitive dysfunction. We investigated the effects of doxorubicin and HDAC6 inhibition on microglia morphology using advanced imaging analysis. To accurately define the microglia phenotype, we performed single-nucleus RNA sequencing of the hippocampus of doxorubicin/ACY-1083-treated, doxorubicin-treated, and control mice. We also investigated the effects of doxorubicin and HDAC6 inhibition on synaptic integrity as a known readout for synaptic and cognitive function 18.

## Materials and Methods

### Mice

Female C57BL/6 J mice (aged 12 weeks at the start of the experiment) were used exclusively due to the low occurrence of breast cancer in males (less than 1%) 23. Mice were obtained from Jackson Laboratories (Bar Harbor, ME) and housed in The University of Texas MD Anderson Cancer Center animal facility on a reversed 12 h light/dark cycle, with free access to food and water. Animals were randomly assigned to treatment groups. All procedures were consistent with the National Institute of Health Guidelines for the Care and Use of Laboratory Animals and were approved by the Institutional Animal Care and Use Committee (IACUC) of M.D. Anderson Cancer Center. Analyses were performed by investigators blinded to treatment.

### Orthotopic Mammary Tumor Model

MMTV-PyMT breast cancer cells were generously provided by Dr. Stephanie Watowich. MMTV-PyMT cells are a murine breast cancer cell line derived from the genetic MMTV-Polyoma Middle T (PyMT) mammary tumor mouse model on the C57BL/6 background, as previously described 24. Briefly, MMTV-PyMT cells were cultured in Dulbecco's modified eagle medium (DMEM) (Thermo Fisher Scientific) containing 10% fetal bovine serum and 1% penicillin-streptomycin. Cells were washed three times with PBS and resuspended in endotoxin-free PBS prior to injection into mice. Ten to twelve-week old female C57BL/6 mice received a unilateral injection of 2.5 × 10^5^ MMTV-PyMT cells in the 4^th^ mammary fat pad. Doxorubicin hydrochloride (5 mg/kg/week, Pfizer, New York, NY) or PBS was administered intraperitoneally beginning 4 weeks after injection with MMTV-PyMT cells (n = 4 mice/group). Doxorubicin treatment occurred weekly for 4 weeks, for a total of 20 mg/kg. Tumor length and width were measured biweekly with electronic calipers. Mice were euthanized when tumors reached 15 mm in any direction or when ulceration >2 mm occurred.

### Drug Administration

Mice were treated with doxorubicin hydrochloride (5 mg/kg/week, Pfizer, New York, NY) or PBS intraperitoneally for 4 weeks, followed by 1 week of rest. Mice were then treated with the blood-brain barrier permeable HDAC6 inhibitor ACY-1083 (10 mg/kg/day, Regenacy Pharmaceuticals, Waltham, MA) or vehicle intraperitoneally daily for 2 weeks. ACY-1083 was dissolved in 20% 2-hydroxypropyl-B-cyclodextrin (Sigma-Aldrich, St. Louis, MO) + 0.5% hydroxypropyl methylcellulose (Spectrum Chemical, Gardena, CA) in milliQ water.

### Behavioral Testing

Cognitive function in female mice (n = 8-16 mice/group) was evaluated using the puzzle box test (PBT) and novel object/place recognition tests (NOPRT) 25, 26. The tests were performed starting 4 weeks after the final dose of ACY-1083 in order to allow for rest, habituation and to evaluate long-term cognitive function.

The PBT was performed as previously described with slight modifications 25. The testing area consisted of a white box divided into two compartments by a black barrier: a brightly lit start zone (58 cm × 28 cm) and a smaller, dark goal box (15 cm × 28 cm) was connected by a 4 cm-wide tunnel. The test consisted of 11 trials at 3 levels of difficulty over the course of 4 consecutive days. In the first easy level of difficulty (training, days 1-2 and trials 1-4) mice were introduced to the brightly lit start box with a connecting tunnel to the dark goal box. In the intermediate level of difficulty (training, days 2-3 and trials 5-7), the tunnel was obstructed with bedding that the mice must burrow through to reach the goal box. In the difficult trials (testing, days 3-4 and trials 8-11), the tunnel was blocked by a cardboard plug that the mice must manipulate to complete the task. Each trial began when the mouse was placed in the start box, and the duration of time it took for the mouse to reach the goal box was recorded with a maximum of 4 minutes during the testing phase. This task utilizes both spatial and short-term working memory regarding the position of the tunnel and recognition of the plug, as well as complex problem-solving in order to remove the plug using their teeth and front paws.

The NOPRT evaluates spatial working memory and was performed as previously described 18. A testing arena (46.99 cm × 25.4 cm) was set up with two identical objects placed on the same side of the arena. In the training phase, mice were placed in the testing arena for 5 minutes and then returned to their home cage. Thirty minutes later, mice were transferred back to the arena that now contains one familiar object in the same location, and one novel object placed on the opposite end of the arena for the testing phase. Investigative behavior was defined as nose point within 1 cm of the object. The time (T) of investigative behavior toward either object during the 5 min testing phase was evaluated using EthoVision XT 10.1 video tracking software (Noldus Information Technology Inc., Leesburg, VA). Discrimination index was determined as (T_Novel_ - T_Familiar_)/(T_Novel_ + T_Familiar_).

### Immunofluorescence

Mice were euthanized 6 weeks after the last dose of ACY-1083 by CO2 euthanasia and perfused intracardially with ice-cold PBS. The skull was removed to allow access to the brain. Brains were removed transferred to 4% paraformaldehyde. Brains were post-fixed in 4% paraformaldehyde for 24 h, cryoprotected in sucrose, and frozen in optimal cutting temperature compound (Sakura Finetek, Torrance, CA). Coronal brain sections (8 or 20 μm) were blocked with blocking buffer (10% normal goat serum, 2% bovine serum albumin and 0.1% saponin in PBS), followed by incubation with rabbit anti-synaptophysin (1:1000, MilliporeSigma (AB9272), Burlington, MA, n = 7-9 mice/group), rabbit anti-PSD95 (1:1000; Abcam (ab18258) Cambridge, UK, n = 9-14 mice/group), rabbit anti-GFAP (K39) (1:1000; OriGene (AP32987SU-N), Rockville, MD, n = 4 mice/group) or rabbit anti-Iba1 (1:1000; Wako (019-19741), Richmond, VA, n = 4 mice/group for 8 μm sections and n = 5-10 mice/group for 20 μm sections) diluted in antibody buffer (2% normal goat serum, 2% bovine serum albumin and 0.1% saponin in PBS) at 4 °C overnight. Slides were then washed three times with PBS, followed by incubation with Alexa-488 goat anti-rabbit (1:500; Invitrogen (A-21206), Carlsbad, CA) or Alexa-647 goat anti-rabbit (1:500; Invitrogen (A-21245)) at room temperature for 2 h. For negative control sections, primary antibody was omitted. After antibody staining, slides were washed three times with PBS, followed by incubation with DAPI (1:5000; Sigma-Aldrich) for 5 min. Slides were then washed three times with PBS, and sealed with FluorSave Reagent (MilliporeSigma). Regions of interest (ROIs) in the hippocampal CA1 and CA3 regions were imaged using a 40x objective with Nikon A1R Confocal Microscope (Nikon Instruments Inc., Melville, NY, USA). The mean fluorescence intensity in each ROI was quantified using Nikon NIS-Elements Advanced Research (Nikon Instruments Inc.).

### Synaptosome Isolation and Mitochondrial Bioenergetics Analysis

Synaptosomes were isolated as previously described 27. Briefly, one hemisphere of the brain (n = 9 mice/group) was homogenized (10% w/v) into 0.32 M sucrose solution in HEPES buffer using a glass Dounce homogenizer. The lysate was centrifuged at 1000 ×*g* for 10 min at 4 °C. The supernatant was mixed with equal volume of 1.3 M sucrose in HEPES buffer and centrifuged at 20,000 ×*g* for 30 min at 4°C. The synaptosomal pellet was then resuspended in XF media (Agilent Technologies, Santa Clara, CA) supplemented with 5.5 mM glucose, 0.5 mM sodium pyruvate, and 1 mM glutamine. Oxygen consumption rate (OCR) was measured with an XF24 Flux Analyzer (Agilent Technologies). Oligomycin (6 μM), carbonyl cyanide 4-(trifluoromethoxy)phenylhydrazone (FCCP, 6 μM), and rotenone/antimycin A (2 μM each) (Sigma-Aldrich) were injected sequentially during the assay. An assay cycle of 2 min mix, 2 min wait, and 2 min measure was repeated three times for baseline rates and after each port injection. Basal respiration, ATP-linked respiration, maximal and spare respiratory capacity were determined as previously described 27.

### Electron Microscopy

For transmission electron microscopy, synaptosomes were fixed in 2% glutaraldehyde + 2% paraformaldehyde in PBS for over 24 h. Samples were then processed as previously described 18. The samples were polymerized in a 60 °C oven for approximately 3 days. Ultrathin sections were cut in a Leica Ultracut microtome (Leica Microsystems, Wetzlar, Germany), stained with uranyl acetate and lead citrate in a Leica EM Stainer (Leica Microsystems), and examined in a JEM 1010 transmission electron microscope (JEOL USA, Inc., Peabody, MA) at an accelerating voltage of 80 kV. Digital images were obtained using AMT Imaging System (Advanced Microscopy Techniques Corp, Danvers, MA). In total, 26-34 mitochondria were quantified from each group (n = 4 mice/group, with 5 images/mouse). Atypical mitochondria were identified by 2-fold increases in diameter and/or excessive vacuolization (more than 50% translucent). The percentage of atypical mitochondria was calculated for each group.

### Microglia Modeling

Hippocampal microglia were visualized by means of immunohistochemical staining of 20 μm-thick coronal brain sections with rabbit anti-Iba1 (1:1000; Wako (019-19741)) as described above. Z-stack images of the hippocampus were acquired with the Nikon A1R Confocal Microscope (Nikon Instruments Inc.) at 1 μm slice intervals at 40x objective. Images were transformed into 3D models using the image analysis software Imaris 9.0 (Bitplane, Concord, MA) and various parameters were measured: total microglia projection length, full branch level, and Sholl analysis. Total microglia projection length is defined as the sum of the length of each projection from the soma of a microglia. The full branch level is a numerical value that begins at the beginning of a projection at the soma with a value of 1. At each branching point, the dendrite segment with the smaller diameter sequentially increases branch level while the dendrite with a larger diameter maintains the same branch level. Finally, Sholl analysis is a valuable tool to visualize and quantify cellular morphology in a 3D realm. In Sholl analysis, the number of filament intersections per concentric spherical shells are quantified beginning at the soma and distanced at a radius of 1μm apart. The area under the curve was computed with GraphPad Prism 8 (GraphPad, San Diego, CA).

### Single-Nucleus RNA-seq Analysis

Nuclei isolation and single-nucleus RNA sequencing were performed by Singulomics Corporation (Singulomics.com). In summary, bilateral hippocampi from 4 mice/group was flash-frozen and combined. Tissue was then homogenized and lysed with Triton X-100 in RNase-free water for nuclei isolation. The isolated nuclei were purified, centrifuged, resuspended in PBS with RNase Inhibitor, and diluted to 700 nuclei/µl for standardized 10x capture and library preparation protocol using 10x Genomics Chromium Next GEM 3' Single Cell Reagent kits v3.1 (10x Genomics, Pleasanton, CA). The libraries were sequenced with Illumina NovaSeq 6000 (Illumina, San Diego, CA).

Preprocessing and quality control: The raw sequencing files were processed with CellRanger 6.0 (10x Genomics). The sequencing was mapped to the mouse genome (mm10) with the option include-introns and generated gene count matrices. The downstream analysis was performed with the R package Seurat 4.0 28. For all the samples, we filtered out the nuclei with <200 genes and >5% mitochondrial genes. The final filtered matrix for 3 samples contained 26823 nuclei and 18961 genes.

Cell type clustering and dimensionality reduction: Each sample was processed separately and then integrated to identify the cell types. The filtered matrix was log-normalized and identified the top 2000 variable genes for each sample using the FindVariableFeatures function. The integration anchors were identified through FindIntegrationAnchors function with the parameter dims = 1:20. To integrate all the samples, the IntegrateData function was applied with parameter dims = 1:20. The integrated data was scaled, and clusters were identified with a resolution of 0.4. The differential expression genes for each cluster were identified using the Wilcoxon rank-sum test which is available in the FindAllMarkers function. We assigned the cell type based on the expression of the known markers which yielded 11 clusters. Microglia cell type was extracted using the Subset function and analyzed for differential gene expression of PBS vs. doxorubicin and doxorubicin vs. doxorubicin + ACY-1083 comparisons.

### Statistical Analysis

Statistical analyses were performed using GraphPad Prism 8 (GraphPad). Error bars indicate SEM and statistical significance was assessed by either unpaired t-test or two-way ANOVA with Tukey's post-hoc analysis.

## Results

### Doxorubicin induces cognitive dysfunction in a therapeutic dosing model that is reversed by HDAC6 inhibition

**Figure 1:**
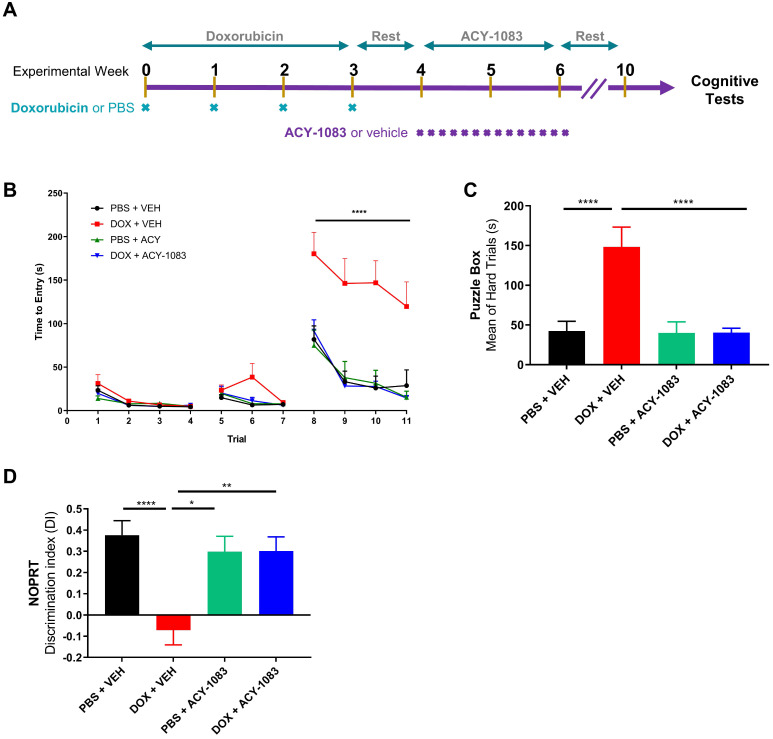
** HDAC6 inhibition with ACY-1083 reverses doxorubicin-induced deficits in executive functioning, working and spatial memory. (A)** Female mice were intraperitoneally treated with doxorubicin (5mg/kg) or PBS, followed by 14 daily administrations of ACY-1083 (10mg/kg) or vehicle. Cognitive function was tested 4 weeks after the final dose of ACY-1083.** (B)** Performance in the puzzle box test with 3 levels of complexity: An open tunnel (easy trials 1-4), a bedding-covered tunnel (intermediate trials 5-7), and a cardboard-plugged tunnel (difficult trials 8-11). Time taken to enter the goal box is recorded. **(C)** The average time taken to enter the goal box during the cardboard-plugged trials (difficult trials 8-11). **(D)** Performance in the NOPRT. The time mice spent interacting with each object was tracked. The discrimination index was calculated as (T_Novel_ - T_Familiar_) / (T_Novel_ + T_Familiar_). Results are expressed as mean ± SEM; n = 8-16 mice/group; Two-way ANOVA with Tukey's post hoc analysis *p ≤ 0.05; **p ≤ 0.01; **** p ≤ 0.0001.

To assess the effects of doxorubicin on cognitive function, doxorubicin hydrochloride (5 mg/kg) was administered intraperitoneally weekly for 4 weeks to female mice (cumulative dose 20 mg/kg). This cumulative dose was shown to effectively reduce tumor growth in a MMTV-PyMT breast cancer mouse model (Figure S1). Following the final dose of doxorubicin, mice were given 1 week of rest. Then, to test the reversal potential of HDAC6 inhibition on cognitive dysfunction, the HDAC6 inhibitor ACY-1083 (10 mg/kg) was administered intraperitoneally daily for a total of 14 days (cumulative dose 140 mg/kg; Figure 1A). This dose was shown to be effective in reversing cognitive dysfunction in a cisplatin-associated model of CICD and did not affect the body weight of mice (Figure S2) 18.

Behavioral testing to evaluate cognitive function began 4 weeks after the last dose of ACY-1083 to allow for rest, habituation and to evaluate the long-term effects of doxorubicin treatment. Mice were first tested using the puzzle box test (PBT), a problem-solving task designed to evaluate executive function and spatial memory 25. In brief, mice were introduced to a brightly lit start box with a connecting tunnel to a dark goal box. Based on light/dark motivation, the time taken to enter the dark goal box was recorded. After 3 days of training with both an unobstructed tunnel (days 1-2, trials 1-4) and a tunnel obstructed with bedding (days 2-3, trials 5-7), mice were tested during the difficult trials (day 3-4, trials 8-11) in which the tunnel was blocked with a cardboard plug that the mice must manipulate to complete the task. Detailed information can be found in the materials and methods section.

During all 4 testing trials (trials 8-11), doxorubicin-treated mice required significantly more time to escape compared to control mice (Figure 1B). Treatment with ACY-1083 completely restored the deficits in spatial memory and executive function observed in doxorubicin-treated mice (Figure 1B). Summation of hard trial averages allows for a clearer representation of doxorubicin-induced deficits in spatial memory and executive function, as well as reversal by treatment with ACY-1083 (Figure 1C).

Next, we employed the novel object/place recognition test (NOPRT) which assesses spatial working memory in mice based on their innate preference for novelty 29. Control mice showed a clear preference for the novel object in a novel location, whereas doxorubicin-treated mice did not show a preference for novelty. (Figure 1D). ACY-1083 treatment restored the preference for novelty in doxorubicin-treated mice, indicating a restoration of spatial and working memory (Figure 1D). Total interaction time spent with the both novel and familiar objects was unchanged between groups (Figure S3). These behavioral results indicate that doxorubicin treatment impaired executive function, spatial memory and working memory in mice long-term. These impairments were completely reversed by the HDAC6 inhibitor ACY-1083.

### Doxorubicin treatment does not alter mitochondrial function or morphology in synaptosomes in a long-term CICD model

**Figure 2:**
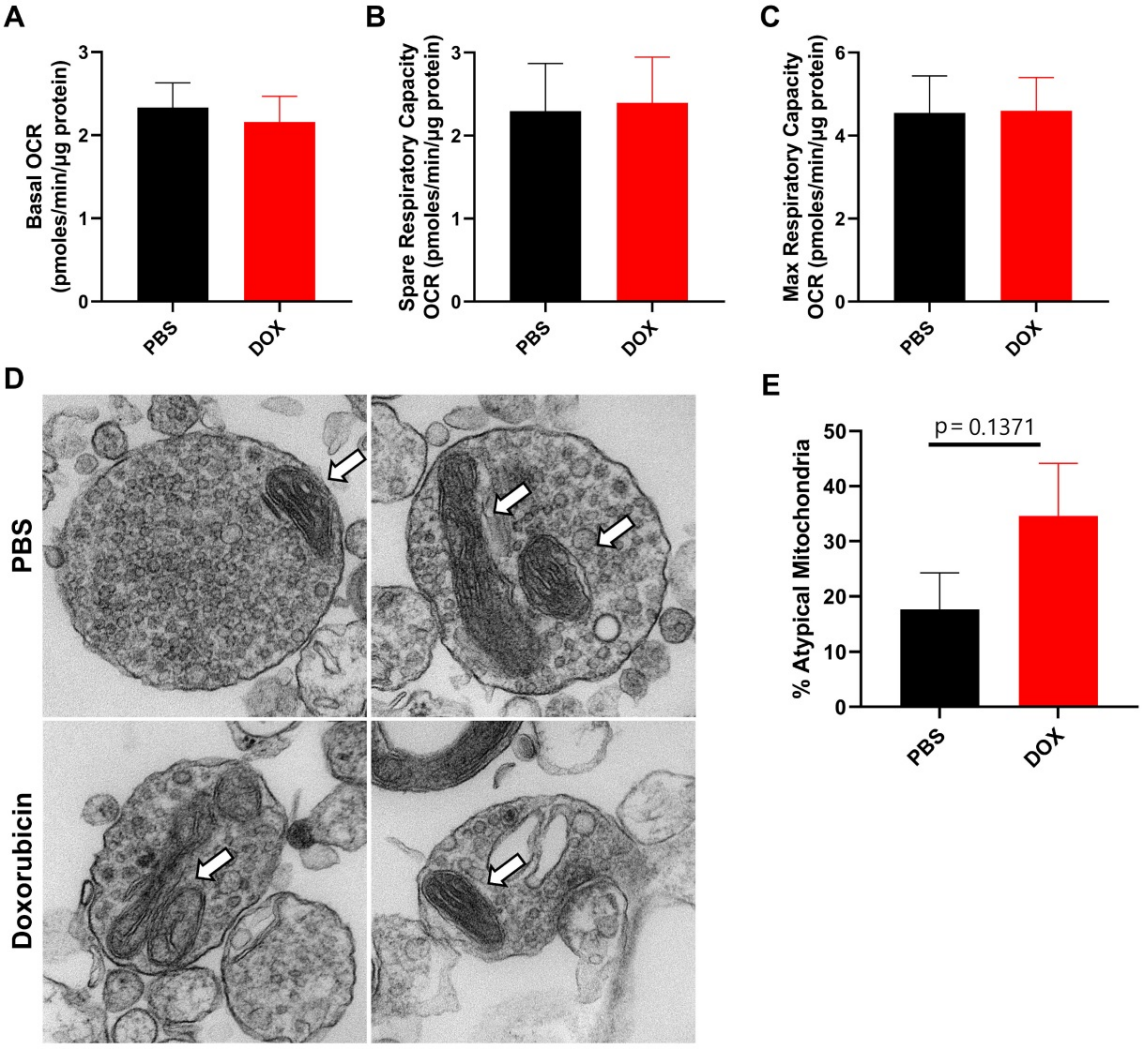
** Doxorubicin treatment does not significantly alter mitochondrial function or morphology in neuronal synaptosomes.** After completion of cognitive tests, synaptosomes were isolated from the brains of mice treated with doxorubicin or PBS. Oxygen consumption rates (OCR) were analyzed in isolated synaptosomes using the Seahorse XFe24 Flux Analyzer. **(A)** Basal respiration, **(B)** spare respiratory capacity, and **(C)** maximum respiratory capacity results are shown. **(D)** Mitochondrial morphology in synaptosomes was assessed using transmission electron microscopy. Arrows indicate mitochondria. **(E)** Percentage of atypical mitochondria was quantified. OCR results are expressed as mean ± SEM; n = 9 mice/group; Unpaired t test. Mitochondrial morphology results are expressed as mean ± SEM; n = 4 mice (26-34 mitochondria)/group; Unpaired t test.

We have previously shown that mitochondrial dysfunction in neuronal synaptosomes is an underlying mechanism in a cisplatin-induced model of cognitive impairment 27. In addition, doxorubicin treatment has previously been shown to alter brain mitochondrial dynamics as assessed 5 days after treatment in rats 30. Therefore, we evaluated CNS mitochondrial function and morphology in neuronal synaptosomes isolated from doxorubicin-treated mice 9 weeks after cessation of treatment. Using the Seahorse XF24 extracellular flux analyzer, we measured the oxygen consumption rate (OCR) to calculate basal, spare and maximum respiratory capacity of mitochondria. Surprisingly, basal, spare and maximum OCR was unaffected by doxorubicin treatment, indicating that long-term doxorubicin-associated CICD is not sustained by impaired mitochondrial function in neuronal synaptosomes (Figure 2A-C). Furthermore, the percentage of atypical mitochondrial morphology, characterized by swollen and/or abnormal cristae structure, was not significantly affected by doxorubicin treatment (Figure 2D-E).

### HDAC6 inhibition restores expression of postsynaptic marker PSD95 in doxorubicin-treated mice

Synaptic integrity is necessary for proper synaptic transmission, synaptic plasticity, and learning and memory 31. To evaluate the effects of doxorubicin and ACY-1083 on synaptic integrity, hippocampal sections were stained with the presynaptic marker synaptophysin and the postsynaptic marker PSD95. The expression level of each marker was quantified in the CA1 and CA3 of the hippocampus.

**Figure 3:**
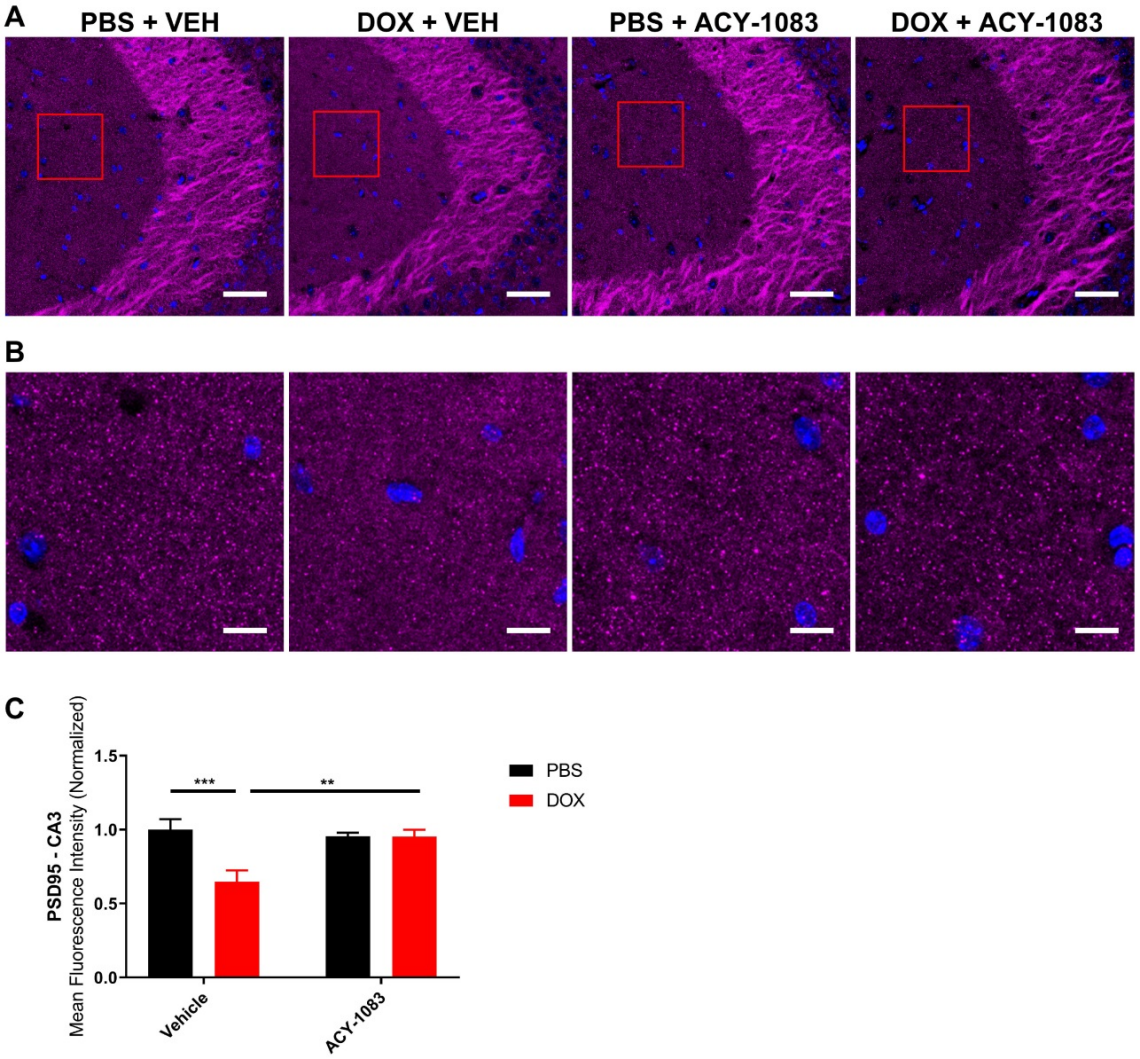
** HDAC6 inhibition with ACY-1083 restores expression of postsynaptic marker PSD95 in doxorubicin-treated mice. (A)** Mouse CA3 hippocampal region stained with PSD95 for different treatment groups; scale bars 50 µm; magnification 40x. **(B)** Higher magnification ROI reveals PSD95+ synaptic puncta; scale bars 10 µm. **(C)** Quantification of the mean fluorescence intensity of PSD95+ puncta. Results are expressed as mean ± SEM; n = 9-14 mice/group; Two-way ANOVA with Tukey's post hoc analysis **p ≤ 0.01; *** p ≤ 0.001.

**Figure 4:**
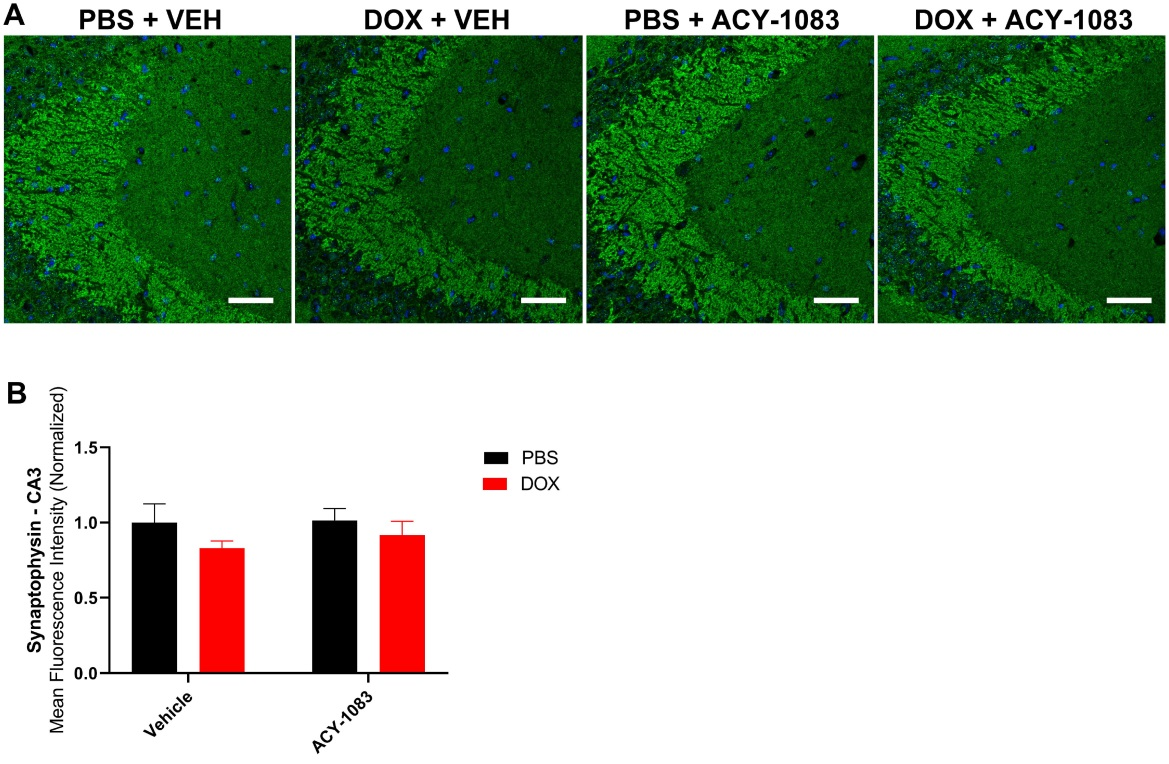
** Doxorubicin does not significantly affect the expression of presynaptic marker synaptophysin. (A)** Mouse CA3 hippocampal region stained with synaptophysin for different treatment groups; scale bars 50 µm; magnification 40x. **(B)** Quantification of the mean fluorescence intensity of synaptophysin staining. Results are expressed as mean ± SEM; n = 7-9 mice/group; Two-way ANOVA with Tukey's post hoc analysis.

In mice treated with doxorubicin, PSD95 expression was significantly decreased in the CA3 (Figure 3A-C) but not the CA1 (Figure S4) of doxorubicin-treated mice compared to the control. This suggests that a decrease in postsynaptic integrity may play a role in sustaining CICD. Treatment with ACY-1083 restored PSD95 expression in the CA3 region of doxorubicin-treated mice, indicating the reversal of postsynaptic density loss (Figure 3A-C). In contrast, there was no significant change in the expression of the presynaptic marker synaptophysin in either the CA3 (Figure 4A-B) or CA1 (Figure S5) of the hippocampus following doxorubicin treatment.

### HDAC6 inhibition reverses doxorubicin-induced reductions in microglial ramification in the hippocampus

**Figure 5:**
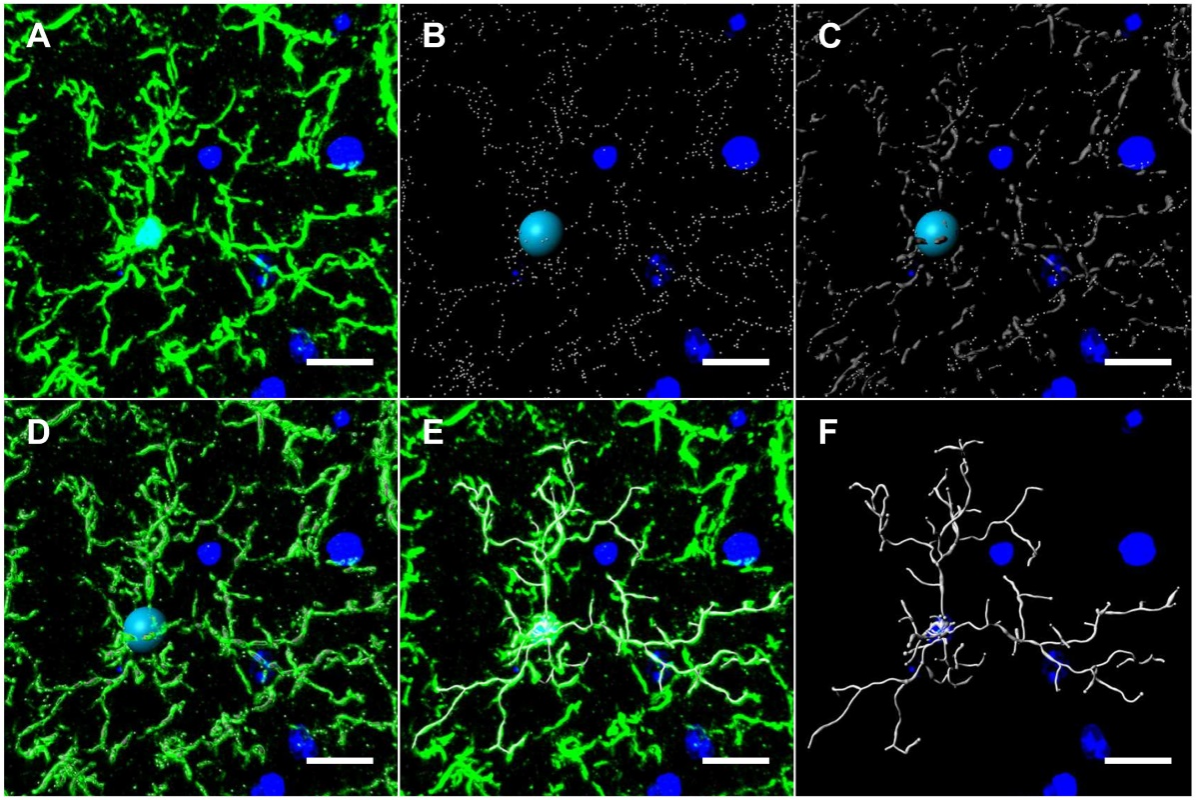
** Microglia modeling workflow using Imaris. (A)** 20 µm-thick mouse hippocampal sections stained with Iba1 were imaged at 1 μm slice intervals at 40x magnification and combined to form a focused, Z-stack image. The isolated Iba1+ GFP channel was 3D modeled using Imaris' filament tracer function. **(B)** Briefly, filament starting points and seed point thresholds were defined by soma diameter size and distal filament diameter, respectively. **(C)** The signal threshold for the detection of filaments was adjusted based on filament diameter size. **(D)** Iba1+ GFP channel overlaid with threshold detection and seed point representations as seen in B and C. **(E)** Iba1+ GFP channel overlaid with 3D filament render. **(F)** Final 3D rendering of Iba1+ microglia, allowing for automated quantification of filament length, branching level and Sholl analysis. Scale bars = 10 µm.

In mouse models, large bolus doses of doxorubicin in the periphery have been shown to induce pro-inflammatory cytokine expression in the CNS, but no study has evaluated therapeutic dosing of doxorubicin, neuroinflammation and its association with long-term CICD 32. Considering the important role glial cells play in mediating neuroinflammation and synaptic pruning, we subsequently quantified GFAP and Iba1 expression in the hippocampus to visualize alterations in astrocytes and microglia, respectively 7. The results indicated no alteration in GFAP expression or astrocyte morphology following doxorubicin treatment (Figure S6). Conversely, Iba1 expression and the length of microglia projections was significantly decreased in the CA1 of the hippocampus following doxorubicin treatment (Figure S6). In order to more accurately define the microglia phenotype in our long-term CICD model, we quantified microglia morphology using 3D cellular modelling and tested whether HDAC6 inhibition with ACY-1083 had the potential to reverse these alterations in conjunction with cognitive dysfunction. Using 20 μm-thick coronal sections, hippocampal microglia were visualized by immunohistochemical staining with an antibody to Iba1. Then, sections were then imaged at 1 μm slice intervals and combined to form a focused, Z-stack image. These images were transformed into 3D models using the image analysis software Imaris (Figure 5), and various parameters were measured: total microglia projection length, full branch level, and Sholl analysis as described in the methods.

**Figure 6:**
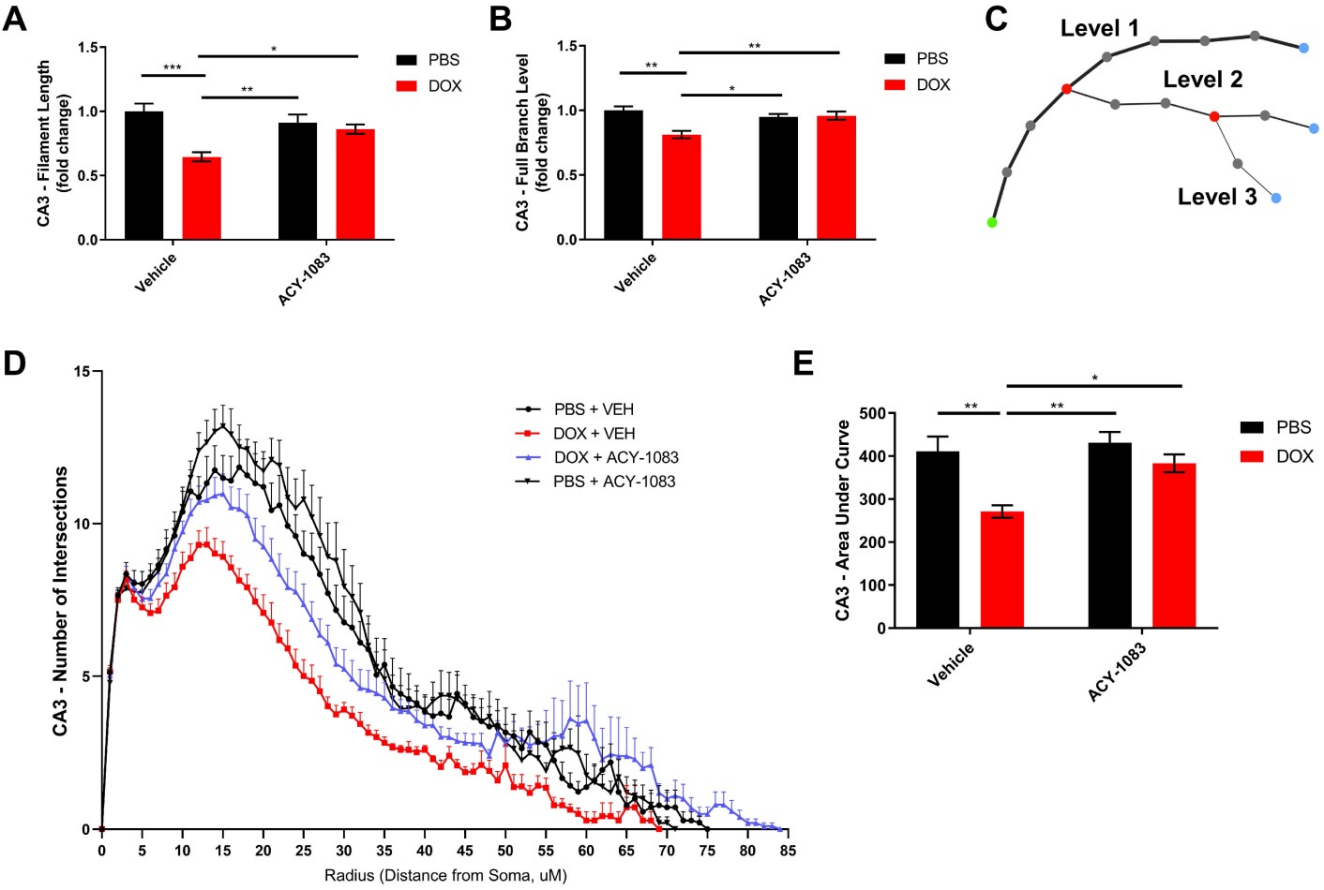
** HDAC6 inhibition with ACY-1083 reverses doxorubicin-induced reductions in microglial ramification in the hippocampus. (A)** Filament length is defined as the sum of the length of each projection from the soma of a microglia. **(B)** Full branch level is the sum of the average branch level of each projection from the soma of a microglia. Branch level is a numerical value that begins at the beginning of a projection at the soma with a value of 1. At each branching point, the filament segment with the smaller diameter sequentially increases branch level while the dendrite with a larger diameter maintains the same branch level. **(C)** Schematic illustration visualizing increasing branch levels. The green dot represents the soma. **(D)** Sholl analysis quantifies the number of projection intersections per concentric spherical shell beginning at the soma and distanced at a radius of 1 μm apart. **(E)** Quantification of the area under the Sholl curve from D. Results are expressed as mean ± SEM; n = 5-10 mice/group; Two-way ANOVA with Tukey's post hoc analysis *p ≤ 0.05; **p ≤ 0.01; *** p ≤ 0.001.

In the CA3 of the hippocampus, the total microglial projection length and the full branch level were significantly decreased in doxorubicin-treated mice indicating a change in morphology that may be indicative of microglia activation. Treatment with ACY-1083 significantly reversed the shortened projection length and decrease in full branch level (Figure 6A-B). Sholl analysis revealed a robust decrease in the number of intersections in doxorubicin-treated mice compared to controls, indicating reduced microglia ramification (Figure 6D). Overall differences between groups were determined using the area under the Sholl curve for each animal, as previously described 33. In the CA3 of doxorubicin-treated animals, the area under the Sholl curve was significantly smaller compared to controls (Figure 6E). Treatment with ACY-1083 significantly increased the area under the Sholl curve, indicating a reversal of altered microglia morphology (Figure 6E). Although reduced microglial ramification was more severe in the CA3 of the hippocampus, similar alterations in microglia morphology were observed in the CA1 region following doxorubicin treatment and reversed with ACY-1083 (Figure S7).

### HDAC6 inhibition reverses doxorubicin-induced transcriptomic alterations in the nucleus of hippocampal microglia

**Figure 7:**
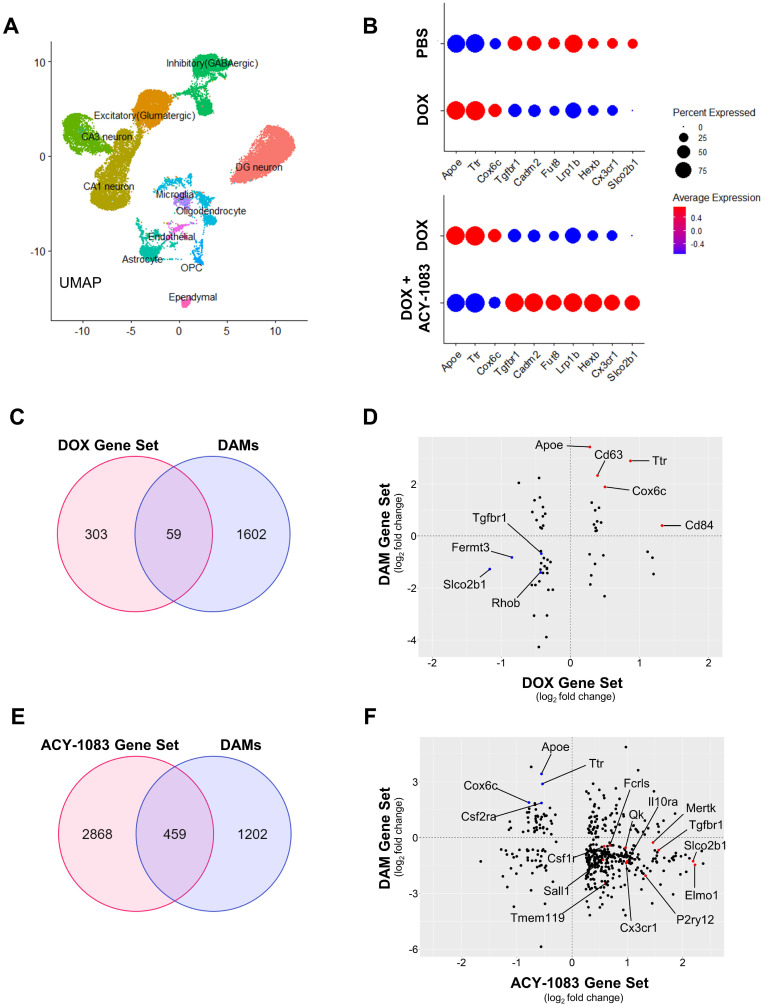
** HDAC6 inhibition with ACY-1083 reverses doxorubicin-induced transcriptomic alterations in the nucleus of hippocampal microglia. (A)** Uniform Manifold Approximation and Projection (UMAP) plot showing 11 cell populations identified from single-nuclear transcriptomes isolated from the hippocampus. **(B)** Dot plot depicting selected microglia-expressed genes associated with neurodegeneration and microglia homeostasis. Dot size encodes percentage of nuclei expressing the gene, while color encodes the average gene expression level per nuclei. **(C)** Venn diagram of genes that were differentially expressed between the DOX Gene Set and DAMs. **(D)** Scatterplot matrix depicting differential expression of microglia-expressed genes in the DOX Gene Set and DAMs. Blue dots indicate selected downregulated genes while red dots indicate selected upregulated genes. **(E)** Venn diagram of genes that were differentially expressed between the ACY-1083 Gene Set and DAMs. **(F)** Scatterplot matrix depicting differential expression of microglia-expressed genes in the ACY-1083 Gene Set and DAMs. Blue dots indicate selected downregulated genes while red dots indicate selected upregulated genes.

While microglia modeling provides evidence of a robust decrease in microglia ramification, it lacks the potential to distinguish between canonical microglia activation and alternative microglia phenotypes (primed, dystrophic, DAMs) due to the morphological similarities between the phenotypes. Therefore, we employed single-nucleus RNA sequencing of hippocampal tissue 6 weeks after the final dose of ACY-1083 to evaluate the transcriptome and clearly define microglia phenotype. After identifying 11 cell populations (Figure 7A), the microglia population was extracted from the integrated data and the differential gene expression of control, doxorubicin-treated, and doxorubicin/ACY-1083-treated mice was calculated. Then, we identified genes that were differentially expressed between doxorubicin-treated and control mice, named DOX Gene Set (362 genes). Genes that were differentially expressed between doxorubicin-treated and doxorubicin/ACY-1083-treated mice were named ACY-1083 Gene Set (3327 genes). After quality control, data from a total of 436 microglia nuclei was analyzed using Seurat 4.0, resulting in the quantification of 3689 expressed genes (Table S1). Finally, in order to identify genes that may contribute to the altered microglia phenotype caused by doxorubicin treatment and subsequent reversal by HDAC6 inhibition, we compared both gene sets to identify common genes that were differentially expressed in both the DOX Gene Set and ACY-1083 Gene Set. We identified the differential regulation of 135 common genes expressed by microglia in both gene sets (Table S1). Of the 135 common genes, 113 genes altered by doxorubicin treatment were reversed following treatment with ACY-1083. Genes upregulated following doxorubicin treatment were associated with neurodegeneration and cellular stress including *Apoe*, *Ttr*, *Cox6c*, *Col6a1*, *Crym*, and *Ntng1*, all of which were downregulated following treatment with ACY-1083. Conversely, doxorubicin treatment led to the downregulation of genes related to microglia homeostasis and synaptic organization including *Slco2b1*, *Fermt3*, *Prickle2*, *Dock3*, *Msn*, *Scamp5*, *Lrp1b*, *Tgfbr1*, *Rhoa*, *Fut8*, *Cadm2*, and *Pdlim5*, all of which were upregulated following treatment with ACY-1083. Interestingly, canonical microglia homeostasis genes that were not affected by doxorubicin treatment but were upregulated by ACY-1083 included *Cx3cr1*, *Tmem119*, *Fcrls*, *Hexb*, *Tgfbr2*, *P2ry12*, *Foxo3*, *Sall1*, and *Gpr34*. A subset of these genes is visualized in Figure 7B. These results indicate that HDAC6 inhibition reverses the altered genetic signature in microglia, while also enhancing homeostatic microglia gene expression, in the hippocampus of doxorubicin-treated mice in our long-term CICD model.

In order to define the microglia phenotype in our long-term CICD model, we compared the DOX Gene Set and ACY-1083 Gene Set to published microglia transcripts from healthy mice and disease models. We found strong similarities between microglia from our long-term CICD model and a unique microglia phenotype recently discovered in AD transgenic mouse brains, termed disease-associated microglia (DAMs) 13. We compared the DOX Gene Set and ACY-1083 Gene Set with genes that were differentially expressed between homeostatic microglia and DAMs (Keren-Shaul, *et al.*, 2017, Table S3; 1661 genes with p<0.05). An overlap of 59 common genes was identified between DAMs and the DOX Gene Set, of which 38 correlated with the expression we observed following doxorubicin treatment (Figure 7C-D and Table S2). This indicates that microglia-expressed genes that were upregulated after doxorubicin treatment were also upregulated in DAM microglia, and vice versa. Notably, we observed a downregulation of microglial homeostasis genes *Tgfbr1*, *Rhob*, *Slco2b1*, and *Fermt3*. We saw an increase in DAM genes associated with oxidative stress and neurodegeneration including *Apoe*, *Cd63*, *Cd84*, *Cd34*, *Cox6c*, *Ttr*, and *Ifnar1* but not phagocytic pathway genes. Following HDAC6 inhibition with ACY-1083, we identified an overlap of 459 genes common to DAMs and the ACY-1083 Gene Set (Figure 7E-F and Table S2). Of these, 337 genes inversely correlated with the expression we observed in our long-term CICD model, indicating a reversal of gene expression and restoration of homeostatic microglia. Notably, we saw an upregulation of the microglial homeostasis genes that were downregulated after doxorubicin treatment: *Tgfbr1*, *Slco2b*, and *Fermt3*. We also observed an increase in microglia homeostasis genes that had not been affected by doxorubicin including *Cx3cr1*, *Tmem119*, *Csf1r*, *Fcrls*, *Mef2a*, *Sall1*, *Maf*, *Gpr34*, *Lrba*, *Med12l*, as well as genes that are typically associated with resolution of inflammation, microglia migration, or purinergic signaling including *Ifngr1*, *Il10ra*, *Tnfaip8*, *Il6ra*, *Pde3b*, *Mef2c*, *Ifngr1*, *Plxna4*, *Selplg*, *Filip1l*, *Pdlim5*, *P2ry12*, and *Entpd1*. We saw robust increases in 8 genes from the PI3K pathway whose activation was shown to promote recovery after injury 34. Interestingly, after HDAC6 inhibition, we saw a large increase in phagocytic pathway genes including *Qk*, *Siglech*, *Elmo1*, *Cd33*, *Itgam*, *Inpp4b*,* Abi3*, and *Nckap1l* which may indicate promotion of repair by active clearance of debris. In addition, we saw an increase in *Mertk*, an engulfment receptor required for the unique non-inflammatory clearance of apoptotic cells called efferocytosis 35. Finally, we saw an upregulation of 22 ubiquitin system genes that play a role in autophagy and phagosome maturation 36. After ACY-1083 treatment, we observed the downregulation of genes that were upregulated following doxorubicin treatment and in DAMs, including neurodegeneration and inflammation-associated genes such as *Apoe*, *Csf2ra*, *Jun*, *Cox6c*, *Tlr7*, and *Ttr*. These results suggest that doxorubicin treatment induces a DAM-like microglia phenotype in our long-term CICD model, which is restored to a homeostatic microglia phenotype following HDAC6 inhibition.

Importantly, Keren-Shaul, *et al*. showed that DAM activation follows a two-step process: a TREM2-independent state, stage 1 DAMs, that are characterized by a loss of microglia homeostasis genes and increase in *Apoe* expression but no increase in phagocytic pathway genes, and a TREM2-dependent transition to stage 2 DAMs in which phagocytic pathway genes are upregulated 13. The lack of *Trem2* expression in our model indicate that *Apoe* upregulation occurred independently of TREM2 signaling which, in addition to the lack of phagocytic pathway gene expression, suggests our microglial phenotype after doxorubicin treatment strongly overlaps with the *stage 1* DAM phenotype.

## Discussion

Cognitive dysfunction following chemotherapy treatment is a severe burden that persists in the lives of many cancer survivors, causing emotional and financial difficulties that significantly lower their quality of life. The goals of our research were to investigate mechanisms of long-term CICD as a result of doxorubicin treatment and whether HDAC6 inhibition using the blood-brain barrier permeable ACY-1083 reverses long-term CICD. Our results demonstrate that doxorubicin treatment induces long-term cognitive dysfunction as assessed by the PBT and the NOPRT. Concurrently, doxorubicin treatment induces long-term morphological and transcriptomic alterations in microglia that suggest a neurodegenerative microglia phenotype closely resembling the stage 1 DAM phenotype described by Keren-Shaul, *et al*. Finally, doxorubicin treatment caused a decrease in postsynaptic PSD95 expression, indicating a decrease in synaptic integrity. Interestingly, doxorubicin treatment did not significantly alter mitochondrial function or morphology in synaptosomes in our long-term CICD model. In contrast, recent research showed that doxorubicin-induced cognitive deficits in male rats was associated with altered mitochondrial dynamics in the brain as assessed 5 days after cessation of treatment 30. Collectively, these findings suggest that mitochondrial dysfunction may be involved in the initiation of CICD but may not be required for long-term CICD as a result of doxorubicin treatment. Following doxorubicin treatment, pharmacological inhibition of HDAC6 with ACY-1083 successfully reversed long-term CICD, restored microglia homeostasis, and restored synaptic integrity. These results suggest that a neurodegenerative microglia phenotype and a decrease in synaptic integrity are an underlying cause of long-term CICD. Previously, we have shown that 14-day inhibition of HDAC6 with ACY-1083 reversed cisplatin-induced cognitive dysfunction by improving mitochondrial bioenergetics 18, 27. Therefore, HDAC6 inhibition exhibits success in reversing CICD with different underlying mechanisms: altered mitochondrial bioenergetics and altered microglia phenotype. Considering the enhanced tumor control HDAC6 inhibition has provided in Phase I and Phase II clinical trials, HDAC6 inhibition proves once again to be a promising therapeutic and disease-modifying intervention for the treatment of CICD.

We previously showed that ACY-1083 is brain penetrant and increases α-tubulin acetylation in a model of cisplatin-induced cognitive dysfunction 18. In the current study, we use the same dosing schedule for the HDAC6 inhibitor and start dosing 7 days after completion of doxorubicin treatment. Given that the terminal plasma half-life of doxorubicin is 20 hours to 48 hours, it is highly unlikely that the previous exposure to doxorubicin would interfere with the inhibitory effect of ACY-1083 on HDAC6 37. In the model of cisplatin-induced cognitive dysfunction, we showed that treatment with a different HDAC6 inhibitor (ACY-1215) that is not brain penetrant was sufficient to reverse cisplatin-induced peripheral neuropathy but not cisplatin-induced cognitive dysfunction 18. This suggests that the beneficial effects of the blood-brain barrier permeable ACY-1083 on cognition take place primarily in the CNS. However, it is possible that an additional peripheral effect of HDAC6 inhibition contributes to the restoration of cognitive dysfunction in our long-term CICD model. It is important to note that the long-term effects of HDAC6 inhibition on cognitive function are not dependent on persistent HDAC6 inhibition considering that the half-life of ACY-1083 is 3.5 hours and behavioral tests were performed 4 weeks after the final dose 38. This suggests that long-term restoration of cognitive function in our model is not just symptom management, but rather a reversal of disease state. Indeed, we propose that following doxorubicin treatment, the HDAC6 inhibitor activates restorative pathways leading to the sustained reversal of changes in microglial phenotype, normalization of synaptic integrity, and restoration of cognitive function.

To our knowledge, this is the first study to use advanced imaging analysis and RNA sequencing to show that doxorubicin treatment induces long-term alterations in microglia morphology and transcriptome that suggest a stage 1 DAM-like phenotype, characterized by a loss of microglia homeostasis genes and an increase in neurodegeneration-associated genes. It is well known that dysregulation of microglial homeostasis is associated with cognitive dysfunction. For instance, persistent microglia activation was shown to predict cognitive decline in the progression of Alzheimer's disease in humans, while elimination of activated microglia restored cognitive function and glial homeostasis in a methotrexane CICD mouse model 39, 40. Although the consequences of dysregulated microglia homeostasis are brain region- and disease-specific, the phenotype is associated with chronic low-grade inflammation and increased ROS production, dysregulation of synapse formation, suppression of neurogenesis, and decreased immune surveillance, all of which may contribute to cognitive impairment 41. Accordingly, our results indicate that doxorubicin treatment leads to a sustained loss of microglia homeostasis genes and increase in neurodegeneration-associated genes in microglia that persist with cognitive dysfunction. These results are consistent with the stage 1 DAM activation state defined by Keren-Shaul, *et al.* in which *Apoe* is upregulated independently of TREM2 signaling and is characterized by an absence in phagocytic pathway genes. Following HDAC6 inhibition, we observed a reversal of the doxorubicin-induced genetic signature including an increase in microglia homeostasis genes and decrease in neurodegeneration-associated genes, as well as an increase in phagocytic pathway genes not observed after doxorubicin treatment. These genetic alterations correlated with a restoration of cognitive function. The increase in microglial phagocytic pathway genes in our long-term CICD model may indicate a promotion of repair by active debris or apoptotic cell clearance that was restricted in our stage 1 DAM-like microglia phenotype as a result of doxorubicin treatment. Interestingly, we did not observe any alterations in astrocyte morphology 9 weeks after doxorubicin treatment contrary to recent research that showed astrocyte reactivity as assessed 5 days after the final dose of doxorubicin in male rats 30. This suggests that while astrocyte reactivity may play a role in the initiation of CICD, it was not involved in sustaining long-term CICD in our model. However, it may be that there are long-term phenotypic alterations in astrocytes that are not observable based on morphology alone. Indeed, we also detected changes in gene expression in astrocytes, oligodendrocytes, and neuronal subpopulations in response to treatment with doxorubicin and the HDAC6 inhibitor. These findings are the subject of further study.

The combined analysis of microglia transcriptome and morphology allows for an accurate definition of microglia phenotype that is unachievable through morphological analysis or gene expression alone. Previously, Allen *et al.* showed that doxorubicin treatment caused microglia activation in the hippocampus of male mice using CD68+ staining and a NanoString mouse immunology panel 42. Although this methodology identifies broadly activated microglia, research suggests that the expression profile of CD68 varies between different stages of microglia activation and, like Iba1, is also expressed by resting microglia 43. In order to accurately characterize the microglial phenotype, simultaneous analysis of gene expression and morphology is required. The current study achieves this by employing single-nucleus RNA sequencing to evaluate the nuclear transcriptome of hippocampal microglia, in addition to 3D morphological analysis, and define the microglia phenotype observed in our long-term CICD model.

Our study only included female mice because the majority of clinical data regarding doxorubicin-induced cognitive dysfunction that form the basis of our study were collected in females treated for breast cancer 44. Recently, sex differences in microglia phenotype have been reported in response to pro-inflammatory stimuli *in vitro* and in models of ischemic stroke *in vivo* 45, 46. These studies showed that male microglia are more reactive and have higher migratory capacity than female microglia 46. Conversely, female microglia exhibit a higher phagocytic capacity and expression of genes related to cellular repair than male microglia 45. Therefore, it remains to be determined whether the findings reported here generalize to males.

The exact mechanism underlying the long-term changes in microglia phenotype in response to treatment with doxorubicin and the HDAC6 inhibitor remains to be determined. HDAC6 mainly deacetylates cytosolic proteins including α-tubulin, HSP90, and peroxiredoxins that regulate the response to oxidative stress 19, 47, 48. Changes in α-tubulin acetylation can affect multiple cellular processes including cell migration and morphology, phagocytosis, and transport of organelles within cells that could all contribute to the restoration of brain function and changes in microglial phenotype that we observe 6 weeks after the last dose of ACY-1083 had been administered to mice previously treated with doxorubicin 49. In addition, the beneficial effects of HDAC6 inhibition on reversing cisplatin-induced peripheral neuropathy are IL-10-dependent (Zhang *et al.,* submitted). IL-10 is an interleukin well-known for the resolution of inflammation and has been shown to prevent pathological microglia phenotypes 50. Additional research during treatment with the HDAC6 inhibitor would be needed to identify the mechanism through which HDAC6 inhibition leads to the long-term restoration of microglial homeostasis we observed in this study.

We also provide evidence that doxorubicin treatment reduces postsynaptic PSD95+ puncta intensity in the hippocampus, supporting previous research that found altered synaptic organization in the hippocampus of doxorubicin-treated mice 51. Interestingly, our results indicate that presynaptic synaptophysin was not affected 9 weeks after doxorubicin treatment. While the mechanism underlying the selective decrease in postsynaptic PSD95 expression is not known, it is well-characterized that PSD95 is highly enriched at glutamatergic synapses 52. Moreover, doxorubicin treatment has been shown to impair glutamate receptors and lead to neuronal toxicity 53, 54. Therefore, doxorubicin may disrupt glutamatergic synapses that contain PSD95. Recently, microglia activation and subsequent C1q-dependent synaptic loss was shown to be correlated with cognitive dysfunction in a model of radiotherapy-induced cognitive dysfunction 55. It is well-documented that microglia mediate synaptic pruning in the postnatal brain by colocalizing with, and engulfing, postsynaptic PSD95 and presynaptic synaptophysin in the hippocampus 56. Interestingly, a mouse model of Alzheimer's revealed that early synapse loss in the hippocampus is specific to PSD95+ puncta, but not synaptophysin+ puncta, and mediated by aberrant synaptic pruning by microglia 57. Therefore, it is very well possible that altered synaptic pruning by microglia with a neurodegenerative phenotype contributed to the decreased PSD95 expression in the hippocampus.

HDAC6 inhibition has previously been shown to prevent the loss of synaptic spine density and PSD95 expression in a mouse model of anesthesia-induced cognitive dysfunction 58. Although the exact mechanism mediating HDAC6 inhibition and the restoration of synaptic integrity is unclear, we observed a robust increase in *Sirpa* expression in microglia following treatment with ACY-1083. *Sirpa* encodes SIRPα, a transmembrane protein that was recently shown to be a negative regulator of synaptic pruning by microglia 59. Loss of *Sirpa* expression in microglia caused aberrant synaptic elimination via increased synaptic phagocytosis and subsequent cognitive impairment 59. Interestingly, HDAC6 inhibition in our model rescues microglia genes associated with phagocytosis of apoptotic cells/debris, while simultaneously upregulating *Sirpa*, which suggests a decrease in the phagocytosis of synaptic elements. In addition to *Sirpa*, we observed the upregulation of *Mertk* after ACY-1083, a gene whose expression is required for the unique non-inflammatory clearance of apoptotic cells called efferocytosis. Efferocytosis is immunologically silent and required to maintain CNS homeostasis 35. The unique mechanisms underlying phagocytosis of debris, phagocytosis of synaptic elements, and efferocytosis may explain how HDAC6 inhibition caused an increase in postsynaptic PSD95 expression while simultaneously increasing phagocytic microglia gene pathways involved in maintaining CNS homeostasis.

The alterations in microglia morphology and PSD95 staining were consistently more significant in the CA3 region than the CA1 region of the hippocampus. These differences may be explained in part by the functional and structural differences between the CA1 and CA3 regions. The CA1 has been shown to mediate temporal associations and maintain short-term memories, while the CA3 is fundamental in the consolidation of spatial and contextual memory, in agreement with our behavioral results 60. Another study showed that novel contextual learning, as evaluated in the NOPRT, is dependent on the CA3 and not the CA1 61. Structurally, recent findings showed that PSD95-associated complexes in the CA1 and CA3 regions represent two distinct neuronal populations 62. It may well be possible that these distinct populations differ in sensitivity to doxorubicin. For example, there is evidence that glutamatergic receptor pathways are over-represented in PSD95-associated complexes in the CA3 region and that doxorubicin preferentially dysregulates excitatory glutamatergic signaling 53, 54, 62. In addition, the CA3 region has been shown to be more sensitive to microglia activation than the CA1 region in models of neuroinflammation, and PSD95 function is regulated in part by microglia 63, 64.

## Conclusions

In conclusion, our results provide evidence that pharmacological inhibition of HDAC6 using the blood-brain barrier permeable drug ACY-1083 successfully reverses long-term CICD as a result of doxorubicin treatment. We show here that therapeutic dosing of doxorubicin leads to CICD and is associated with alterations in microglial transcriptome and morphology that suggests a neurodegenerative microglia phenotype closely resembling the stage 1 DAM phenotype, as well as decreased postsynaptic integrity. Inhibition of HDAC6 reversed CICD and restored microglial homeostasis, which likely contributes to the restoration of postsynaptic integrity. Taken together, our novel findings indicate that treatment with a blood-brain barrier permeable HDAC6 inhibitor shows promise as a pharmaceutical intervention to reverse the microglia-mediated neurotoxic effects of doxorubicin treatment in association with a reversal of CICD. This study has implications in reversing cognitive dysfunction, and therefore improving the quality of life, in breast cancer survivors globally.

## Supplementary Material

Supplementary figures.

Supplementary table 1.

Supplementary table 2.

## Abbreviations

CA1: cornu ammonis 1
CA3: cornu ammonis 3
CICD: chemotherapy-induced cognitive dysfunction
CNS: central nervous system
DAM: disease-associated microglia
DOX: doxorubicin
GFAP: glial fibrillary acidic protein
HDAC6: histone deacetylase 6
IBA1: ionized calcium binding adaptor molecule 1
IL-10: interleukin 10
IP: intraperitoneally
LPS: lipopolysaccharide
MMTV-PyMT: mouse mammary tumor virus-polyoma middle tumor-antigen
NOPRT: novel object/place recognition test
OCR: oxygen consumption rate
PBS: phosphate buffered saline
PBT: puzzle box test
PI3K: phosphoinositide 3-kinases
PSD95: postsynaptic density protein 95
RNA: ribonucleic acid
ROI: regions of interest
SIRPα: signal regulatory protein α
TREM2: triggering receptor expressed on myeloid cells 2
