# Supplementary material for: HDAC6 inhibition reverses long-term doxorubicin-induced cognitive dysfunction by restoring microglia homeostasis and synaptic integrity
Source: Theranostics. 2022 Jan 1;12(2):603–19. doi: 10.7150/thno.67410 (PMC8692908; doi:10.7150/thno.67410)
Supplement: Supplementary file 1 — Supplementary figures. [file thnov12p0603s1.pdf]

Supplementary data

HDAC6 inhibition reverses long-term doxorubicin-induced cognitive dysfunction by restoring microglia homeostasis and synaptic integrity

Blake R McAlpin, Rajasekaran Mahalingam, Anand K Singh, Shruti Dharmaraj, Taylor T Chrisikos, Nabila Boukelmoune, Annemieke Kavelaars, and Cobi J Heijnen

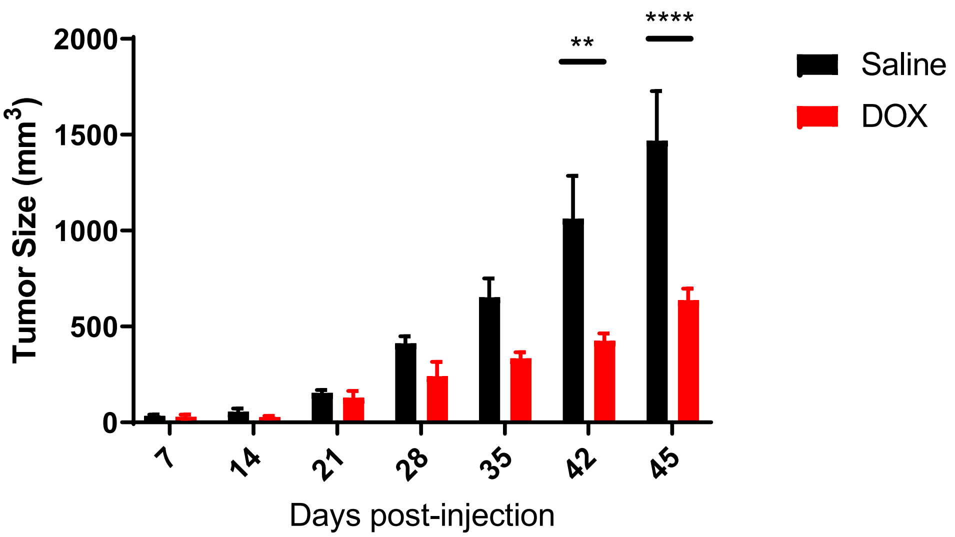

Supplementary Figure 1. Doxorubicin inhibition of tumor growth *in vivo* in a PyMT breast cancer model. On day 0, 2.5 x 10^5 PyMT cells were injected in the 4th mammary fat pad. Doxorubicin treatment began at day 28. Results are expressed as mean ± SEM; n = 4 mice/group; Unpaired t test $^{**}p \leq 0.01$; **** $p \leq 0.0001$.

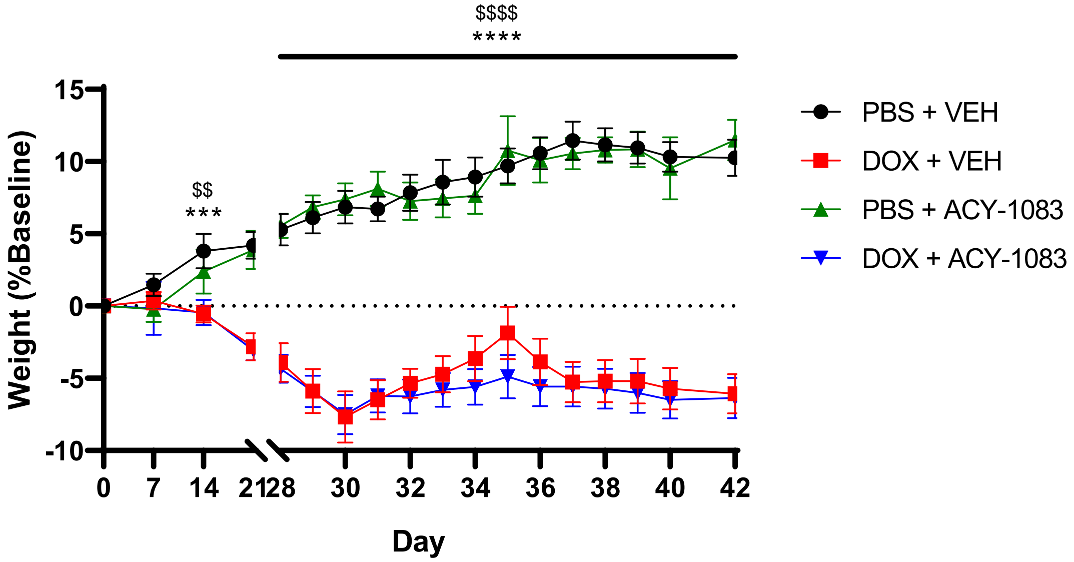

Supplementary Figure 2. Percentage of baseline body weight was recorded. Results are expressed as means ± SEM; n = 5-11 mice/group; Two-way ANOVA with Tukey's post hoc analysis; ***p ≤ 0.001; **** p ≤ 0.0001 PBS/VEH vs. DOX/VEH and PBS/VEH vs DOX/ACY-1083; $$p ≤ 0.01; $$$$p ≤ 0.0001 PBS/ACY-1083 vs. DOX/VEH and PBS/ACY-1083 vs. DOX/ACY-1083.

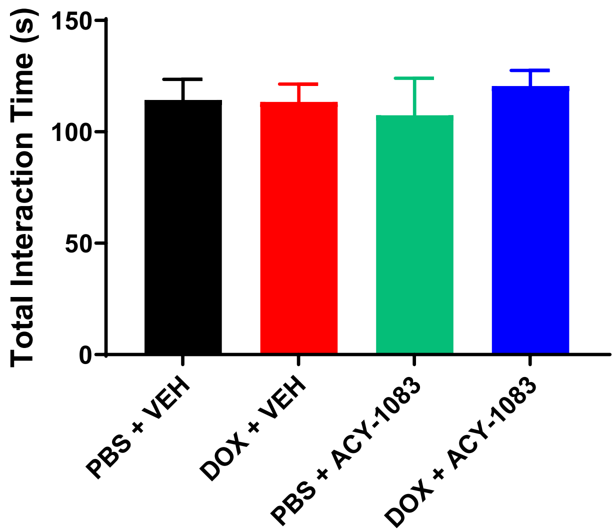

Supplementary Figure 3. Total interaction time with both novel and familiar objects in the novel object place recognition test. Results are expressed as mean ± SEM; n = 8-16 mice/group; Two-way ANOVA with Tukey's post hoc analysis.

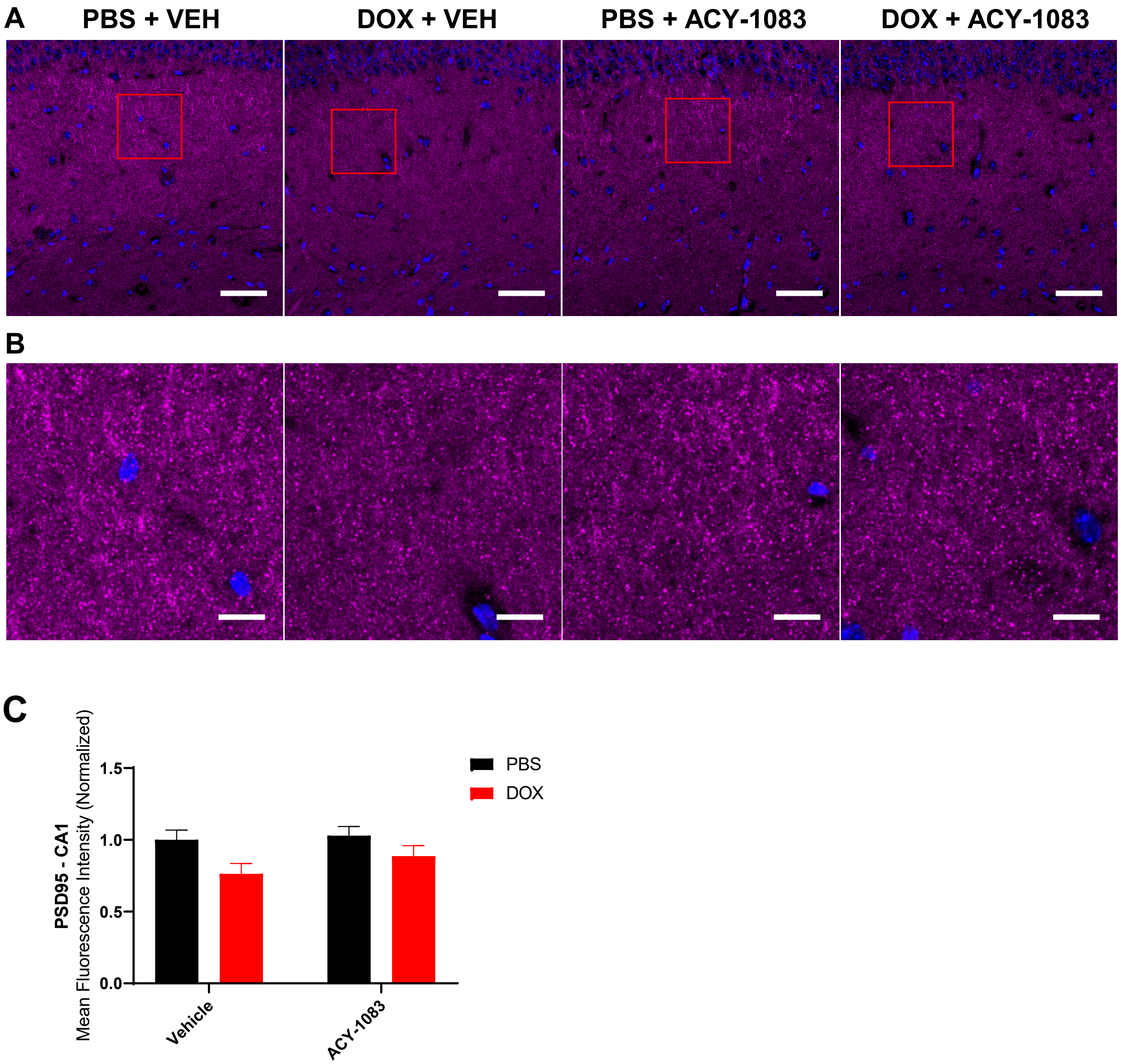

Supplementary Figure 4. PSD95 expression in the CA1 of the hippocampus. **(A)** Mouse CA1 hippocampal region stained with PSD95 for different treatment groups; scale bars 50 µm; magnification 40x. **(B)** Higher magnification ROI reveals PSD95+ synaptic puncta; scale bars 10 µm. **(C)** Quantification of the mean fluorescence intensity of PSD95+ puncta. Results are expressed as mean ± SEM; n = 9-14 mice/group; Two-way ANOVA with Tukey's post hoc analysis.

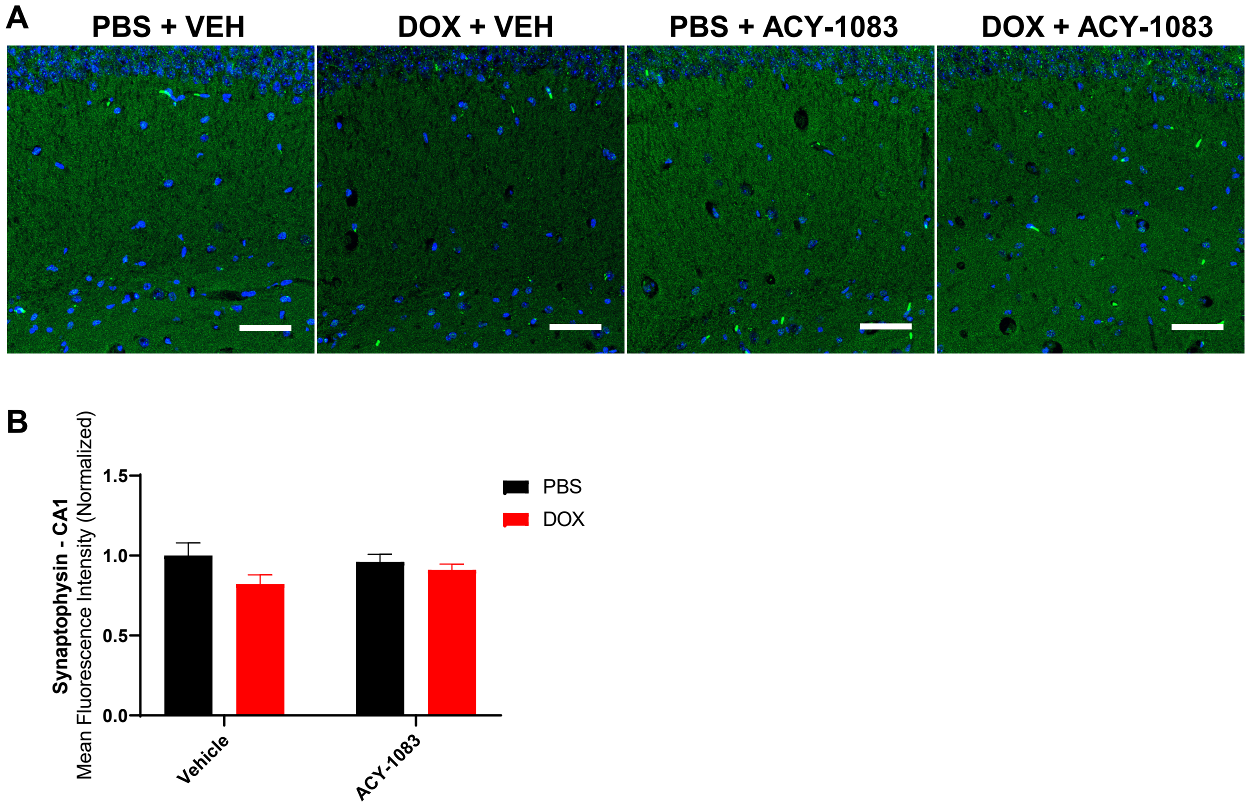

Supplementary Figure 5. Synaptophysin expression in the CA1 of the hippocampus. **(A)** Mouse CA1 hippocampal region stained with synaptophysin for different treatment groups; scale bars 50 µm; magnification 40x. **(B)** Quantification of the mean fluorescence intensity of synaptophysin staining. Results are expressed as mean ± SEM; n = 7-9 mice/group; Two-way ANOVA with Tukey's post hoc analysis.

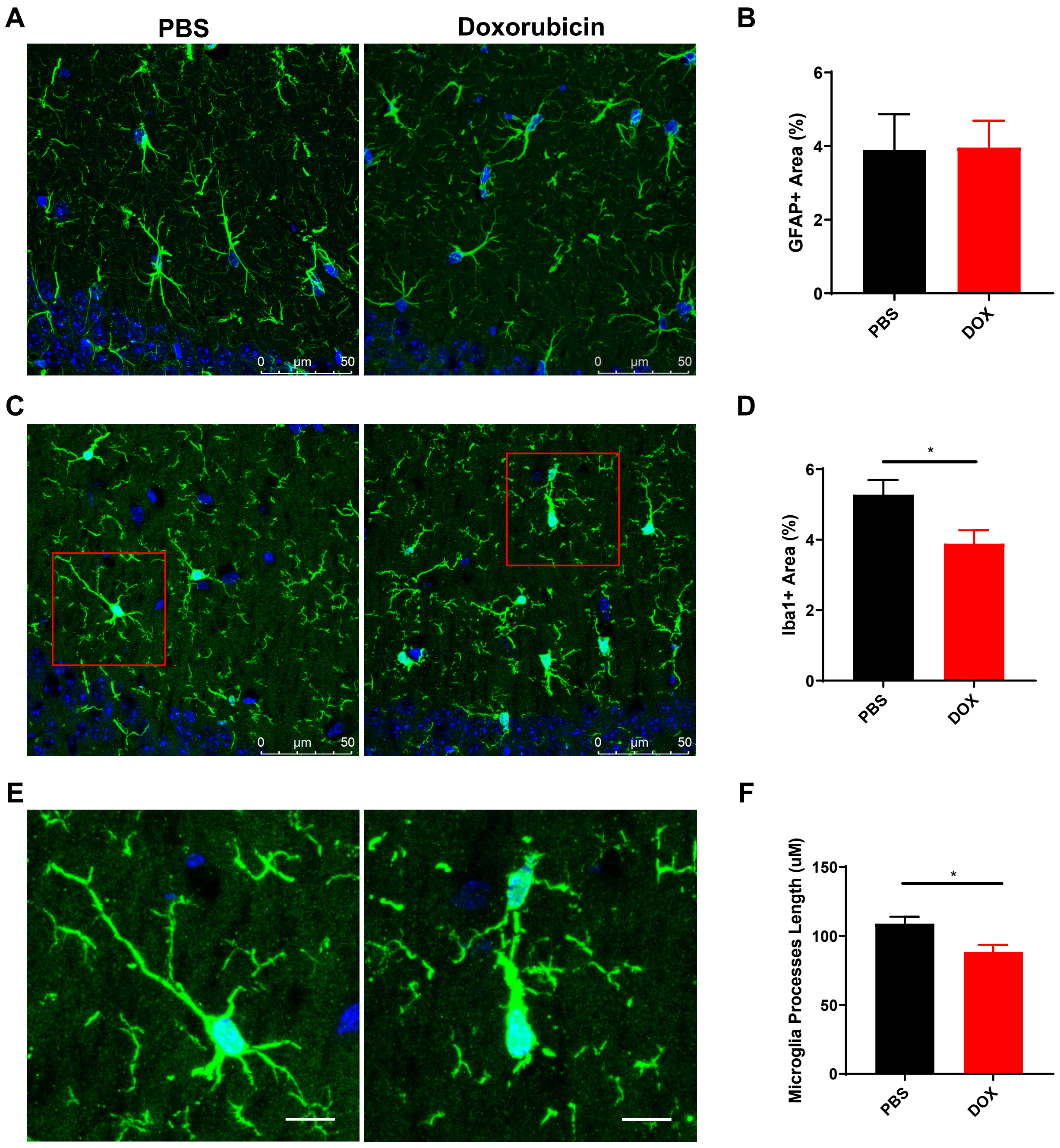

Supplementary Figure 6. (A) 8 µm-thick mouse hippocampal sections stained with GFAP to visualize astrocyte morphology; scale bars 50 µm. **(B)** Percentage of GFAP+ staining area was unchanged following doxorubicin treatment. **(C)** 8 µm-thick mouse hippocampal sections stained with Iba1 to visualize microglia morphology; scale bars 50 µm;

magnification 40x. **(D)** Percentage of Iba1+ staining area was decreased following doxorubicin treatment. **(E)** Higher magnification ROI reveals an altered microglia morphology and **(F)** significant decrease in microglia projection length; scale bars 10 μm. Results are expressed as mean ± SEM; n = 4 mice/group; Unpaired t test $*p \leq 0.05$.

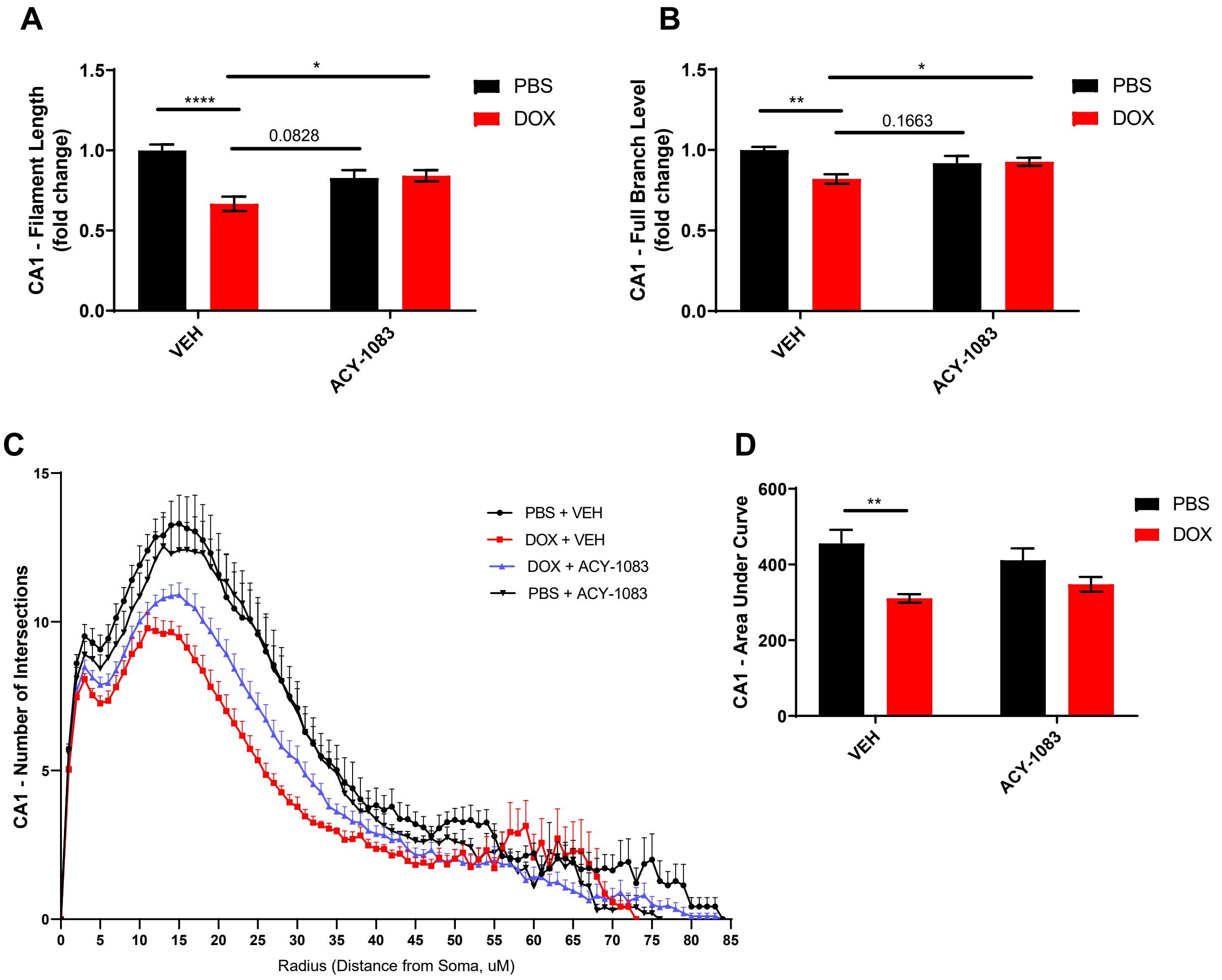

Supplementary Figure 7. ACY-1083 reverses doxorubicin-induced alterations in microglia morphology in the CA1 region of the hippocampus. **(A)** Filament length of CA1 microglia. **(B)** Full branch level of CA1 microglia. **(C)** Sholl analysis of CA1 microglia. **(D)** Quantification of area under the Sholl curve from C. Results are expressed as mean ± SEM; n = 5-10 mice/group; Two-way ANOVA with Tukey's post hoc analysis $*p \leq 0.05$; $**p \leq 0.01$; $****\ p \leq 0.0001$.
